# Data on the I–V characteristics related to the SM55 monocrystalline PV module at various solar irradiance and temperatures

**DOI:** 10.1016/j.dib.2019.104527

**Published:** 2019-09-16

**Authors:** Yassine Chaibi, Maria Malvoni, Amine Allouhi, Salhi Mohamed

**Affiliations:** a ENSAM, Moulay Ismail University, El Mansour, BP 15290, Meknes, Morocco; b School of Electrical and Computer Engineering, National Technical University of Athens, Greece; c Ecole Supérieure de Technologie de Fès, U.S.M.B.A, Route D'Imouzzer, BP 2427, Fez, Morocco

**Keywords:** PV cell modelling, Parameters extraction methods, I–V characteristics, Manufacturer data

## Abstract

The presented data are related to the article “Solar Irradiance and Temperature Influence on the Photovoltaic Cell Equivalent-Circuit Models” (Chaibi et al., 2019). Data include the open-circuit voltage, the short-circuit current and the output power of the Shell SM55 mono-crystalline Photovoltaic (PV) Solar Module obtained from a PV panel modelling based on the single-diode and the double-diode circuit models, coupled with Chaibi and Ishaque parameter extraction techniques (Chaibi et al., 2018, Ishaque et al., 2011). The I–V curves as simulation results are provided at various levels of solar irradiance and temperature.

**Table:** Specifications Table

Subject	Energy Engineering and Power Technology
Specific subject area	Photovoltaic Solar Energy
Type of data	Table Excel file
How data were acquired	Numerical simulations in MATLAB environment of the PV module modelling based on the single-diode and double-diode equivalent circuit models. Graphical curve fitting method using GraphClick software.
Data format	Raw and Simulated data
Parameters for data collection	Different level of solar irradiance (200, 400, 600, 800 and 1000 W/m^2^) and temperature (20, 30, 40, 50 and 60 °C) were considered to collect data related to output power, open-circuit voltage and short circuit-current of the SM55 Module.
Description of data collection	The output power, open-circuit voltage and short circuit-current of the SM55 Module were collected by the implementation of single-diode and double-diode models as the equivalent circuit models in Matlab environment and compared with the corresponding values from datasheet curves for different irradiance levels and temperature.
Data source location	Institution: ENSAM - Moulay Ismail University City: Meknes Country: Morocco
Data accessibility	Data are provided in supplementary materials with this article.
Related research article	The presented data are related to the article Y. Chaibi, A. Allouhi, M. Malvoni, M. Salhi, R. Saadani « Solar Irradiance and Temperature Influence on the Photovoltaic Cell Equivalent-Circuit Models, Solar Energy, 188 (2019), 1102–1110, https://doi.org/10.1016/j.solener.2019.07.005.

**Table:** 

**Value of the Data** • The simulation data related to open-circuit voltage, short-circuit current and output power can be applied to analyse the influence of the equivalent circuit models in the electrical parameters determination. • The I–V curves represent a support to investigate the changes of the electrical performance of the monocrystalline PV module at various levels of solar irradiance and temperature. • The I–V coordinates can be very useful to the research community for the validation of new techniques related to PV module modelling.

## 1 Data

This paper presents the numerical data related to open-circuit voltage, short-circuit current and maximum power obtained from the modeling of monocrystalline SM55 module under changes in the solar irradiance and the temperature. Such electrical parameters are depicted in the Table 2, Table 3, Table 4, Table 5, Table 6, Table 7. The single-diode model (SDM) and the double-diode model (DDM) are implemented as solar cell equivalent circuit models combined with Chaibi et al. [2] and Ishaque et al. [3] techniques respectively to determinate the related unknown parameters. The I–V curve data intended as simulation outcomes are provided in the supplementary file that includes the I–V characteristics at 200, 400, 600, 800 and 1000 W/m^2^ of irradiance and 20, 30, 40, 50 and 60 °C of temperature.

**Table 1:** Datasheet parameters of Shell SM55 Module at STC (Standard Test Conditions).

Parameters	Mono-Si SM55
P_m_ [W]	55
V_m_ [V]	17.4
I_m_ [A]	3.15
V_oc_ [V]	21.7
I_sc_ [A]	3.45
K_I_ [%/K]	0.04
K_v_ [%/K]	−0.35
N_cell_	36

**Table 2:** Open-circuit voltage of the Mono-Si SM55 PV panel using various equivalent circuit models and extracted values from datasheet curves for different irradiances, T = 25 °C.

	Datasheet [V]	SDM [V]	DDM [V]
200 W/m^2^	19.69	19.11	19.60
400 W/m^2^	20.52	20.22	20.35
600 W/m^2^	21.02	20.88	20.75
800 W/m^2^	21.33	21.34	21.04
1000 W/m^2^	21.62	21.70	21.26

**Table 3:** Short-circuit current of the Mono-Si SM55 PV panel using various equivalent circuit models and extracted values from datasheet curves for different irradiances, T = 25 °C.

	Datasheet [A]	SDM [A]	DDM [A]
200 W/m^2^	0.690	0.690	0.689
400 W/m^2^	1.380	1.380	1.378
600 W/m^2^	2.070	2.070	2.067
800 W/m^2^	2.760	2.760	2.757
1000 W/m^2^	3.450	3.450	3.446

**Table 4:** Output power of the Mono-Si SM55 PV panel using various equivalent circuit models and extracted values from datasheet curves for different irradiances, T = 25 °C.

	Datasheet [W]	SDM [W]	DDM [W]
200 W/m^2^	9.88	9.48	9.20
400 W/m^2^	21.06	20.31	20.75
600 W/m^2^	32.45	31.58	32.29
800 W/m^2^	43.77	43.11	43.07
1000 W/m^2^	55.07	54.81	54.81

**Table 5:** Open-circuit voltage of the Mono-Si SM55 PV panel using various equivalent circuit models and extracted values from datasheet curves for different temperatures, λ = 1000 W/m^2^.

	Datasheet [V]	SDM [V]	DDM [V]
20 °C	22.16	22.09	22.64
30 °C	21.40	21.31	20.88
40 °C	20.61	20.52	20.11
50 °C	19.88	19.73	19.34
60 °C	19.1	18.94	19.00

**Table 6:** Short-circuit current of the Mono-Si SM55 PV panel using various equivalent circuit models and extracted values from datasheet curves for different temperatures, λ = 1000 W/m^2^.

	Datasheet [A]	SDM [A]	DDM [A]
20 °C	3.453	3.443	3.439
30 °C	3.459	3.457	3.453
40 °C	3.472	3.471	3.467
50 °C	3.484	3.484	3.481
60 °C	3.497	3.498	3.495

**Table 7:** Output power of the Mono-Si SM55 PV panel using various equivalent circuit models and extracted values from datasheet curves for different temperatures, λ = 1000 W/m^2^.

	Datasheet [W]	SDM [W]	DDM [W]
20 °C	56.54	56.13	56.01
30 °C	53.80	53.47	53.61
40 °C	51.19	50.81	51.19
50 °C	48.56	48.14	48.76
60 °C	46.01	45.48	46.01

## 2 Experimental design, materials, and methods

The technical specifications of the monocrystalline SM55 module as given by the manufacturer are presented in Table 1 [4].

The assessment of the changes of voltage, current and output power of a PV module requires an Equivalent Circuit Solar Cell Model. The single-diode model (SDM) and the double-diode model (DDM) are the most popular PV cell equivalent-circuit models to evaluate the electrical performance of solar PV cell. They include unknown parameters which need to be determined accurately. The methods proposed by Chaibi et al. [2] and by Ishaque et al. [3] are implemented to extract the parameters of SDM and DDM respectively. More details about the implementation of the SSD and the DDM combined with Chaibi et al. [2] and Ishaque et al. [3] parameters extraction methods can be found in [1].

Graphical curve fitting method is implemented by using the graph digitizer software GraphClick [5] in order to retrieve a series of data point with the best fit for the I–V curves provided in the manufacturer datasheet.

The output power, open-circuit voltage and short circuit-current of the SM55 Module, obtained from the manufacturer I–V curves at various level of solar irradiance and temperature are depicted in the Table 2, Table 3, Table 4, Table 5, Table 6, Table 7 They include also the electrical parameters obtained from the SDM and DDM simulations.
